# Cognitive Functioning in Clinically Stable Patients with Bipolar Disorder I and II

**DOI:** 10.1371/journal.pone.0115562

**Published:** 2015-01-23

**Authors:** Timea Sparding, Katja Silander, Erik Pålsson, Josefin Östlind, Carl Sellgren, Carl Johan Ekman, Erik Joas, Stefan Hansen, Mikael Landén

**Affiliations:** 1 Institute of Neuroscience and Physiology, Department of Psychiatry and Neurochemistry, the Sahlgrenska Academy, University of Gothenburg, Gothenburg, Sweden; 2 Department of Medical Epidemiology and Biostatistics, Karolinska Institutet, Stockholm, Sweden; 3 Department of Clinical Neuroscience, Karolinska Institutet, Stockholm, Sweden; 4 Department of Psychology, University of Gothenburg, Gothenburg, Sweden; University of Hertfordshire, UNITED KINGDOM

## Abstract

**Objectives:**

Bipolar disorder is accompanied by cognitive impairments, which persists during euthymic phases. The purpose of the present study was to identify those neuropsychological tests that most reliably tell euthymic bipolar patients and controls apart, and to clarify the extent to which these cognitive impairments are clinically significant as judged from neuropsychological norms.

**Methods:**

Patients with bipolar disorder (type I: *n* = 64; type II: *n* = 44) and controls (*n* = 86) were examined with a comprehensive neuropsychological test battery yielding 47 measures of executive functioning, speed, memory, and verbal skills. Multivariate analysis was used to build a model of cognitive performance with the ability to expose underlying trends in data and to reveal cognitive differences between patients and controls.

**Results:**

Patients with bipolar disorder and controls were partially separated by one predictive component of cognitive performance. Additionally, the relative relevance of each cognitive measure for such separation was decided. Cognitive tests measuring set shifting, inhibition, fluency, and searching (e.g., Trail Making Test, Color-Word) had strongest discriminating ability and most reliably detected cognitive impairments in the patient group.

**Conclusions:**

Both bipolar disorder type I and type II were associated with cognitive impairment that for a sizeable minority is significant in a clinical neuropsychological sense. We demonstrate a combination of neuropsychological tests that reliably detect cognitive impairment in bipolar disorder.

## Introduction

Even though recurrent episodes of depression and mania are the hallmarks of bipolar disorder, an association between bipolar disorder and cognitive impairment has also repeatedly been described, foremost regarding executive function, attention, processing speed, and verbal- and episodic memory [1–3]. Yet, it is undecided whether cognitive impairment is a general trait of bipolar disorder. Bipolar patients with a history of psychotic symptoms have been suggested to be more cognitively impaired than non-psychotic patients [4]. Also, earlier studies disagree as to whether bipolar disorder I and II differ with respect to cognitive impairment. Some studies report no differences between these subtypes [5, 6], whereas others suggest that bipolar disorder I patients perform poorer than patients with bipolar disorder type II [7–9].

A recent meta-analysis of cognition in bipolar disorders showed a large degree of inconsistency across studies and that case-control differences were smaller than previously thought [10]. Even though cognitive impairment during euthymic periods are thought to contribute to the bipolar patient’s difficulties in everyday occupational and social functioning [11], the clinical significance of case-control differences in cognitive function remains to be determined.

The magnitude of these case-control differences across studies assessing cognitive functioning has guided the choice of cognitive tasks recommended by the International Society of Bipolar Disorders [12]. However, studies of cognitive functioning have an innate difficulty concerning the choice of appropriate statistical method. While not yet on par with ‘omics’ datasets, modern neuropsychological test batteries yield dozens of inter-correlated measures, which demand efficient statistical tools to extract robust trends and to avoid false-positives. Here, we therefore used orthogonal partial least-squares to latent structures (OPLS) [13] in order to characterize cognition in bipolar disorder. OPLS in its discriminant analysis form (OPLS-DA) splits the systematic variation in a dataset (47 neuropsychological test results for each participant in the present case) into two parts. One is predictive of class membership (i.e., bipolar I, bipolar II, healthy controls in the present case) and the other is uncorrelated or orthogonal to the classes. This partitioning greatly facilitates model interpretation and identifies the combination of neuropsychological variables that separate pre-defined groups.

The aims of this study was (i) to identify those neuropsychological tests that most reliably tell euthymic bipolar patients and healthy controls apart; (ii) to clarify the extent to which these cognitive impairments are clinically significant in a neuropsychological sense as opposed to merely statistically different from a healthy control group; (iii) to elucidate if patients with bipolar disorder I and II are cognitively dissimilar; (iv) to elucidate if the degree of cognitive impairment has bearing on psychosocial and clinical variables. We studied these questions in a clinical cohort of euthymic bipolar patients and matched healthy controls that had completed a comprehensive neuropsychological test battery. Some parts of the neurocognitive data from patients and controls enrolled in this study have been used in a previous study that used univariate analysis to compare cases and controls [6]. Here, we the OPLS-DA procedure that we hypothesized would uncover information on neurocognitive performance that is unavailable when using univariate analyses.

## Methods

### Subjects

The project was approved by Stockholm Regional Ethical Review Board and all participants consented to take part in the study. The data derive from St. Göran Bipolar Project, which has been described in detail previously [14–16]. Briefly, patients were recruited from the Affective Center at Northern Stockholm Psychiatry. The diagnostic instrument for bipolar disorder was the Affective Disorder Evaluation (ADE), which is a semi-structured interview that includes adapted versions of the mood and psychosis modules of the Structured Clinical Interview for DSM-IV (SCID), and was developed for the Systematic Treatment Enhancement Program of Bipolar Disorder (STEP-BD) project [17]. These diagnoses were confirmed by a consensus panel of experienced clinicians whereby a best estimate diagnosis was reached. Thus 64 participants met the criteria for bipolar I disorder and 44 for bipolar II disorder (*n* = 44). Co-morbid psychiatric disorders were screened for by using the Mini International Neuropsychiatric Interview (M.I.N.I.) [18]. To screen for alcohol and substance abuse, the self-report questionnaires Alcohol Use Disorders Identification Test (AUDIT) [19] and Drug Use Disorders Identification Test (DUDIT) [20] were used. In addition to information regarding age, sex, and level of education, records were available concerning age at first symptom, age at first psychosis (if any), lifetime history of psychosis, number of affective episodes, electroconvulsive treatments, number of sick leave-days the last year, and primary income source. Data on medication was collected at the baseline diagnostic assessment and the somatic examination. Drug information was therefore harvested from the date closest in time to the date of testing. The severity of bipolar disorder was rated using the Clinical Global Impression (CGI) [21] rating scale. Overall functioning was assessed with GAF [22].

Euthymia was defined by Montgomery Åsberg Depression Rating Scale (MADRS) [23] and the Young Ziegler Mania Rating Scale (YMRS) [24] scores of <14 at the time of neuropsychological testing (Table 1).

**Table 1:** Summary of demographic and clinical characteristics in patients with bipolar disorder I (n = 64) and bipolar disorder II (n = 44).

	**bipolar I^a^**		**bipolar II^b^**	
	**mean**	**s.d.**	**mean**	**s.d.**
**Age**	38	14	35	12
**Education**	3.7	1.1	3.9	1.2
**% female**	52		55	
**MADRS**	3	3	3	3
**YMRS**	1	2	2	2
**GAF**	69	11	68	10
**Illness debut**	19	9	18	11
**No. of episodes**	19	26	18	18
**Sick-leave days previous year**	121	164	116	169
**In work (%)**	58		67	
**History of psychosis (%)**	73		7	
**Age at first psychosis**	27	11	26	9
**Antipsychotic medication (%)**	32		11	
**Lithium (%)**	68		48	
**Antidepressant (%)**	31		41	
**Anticonvulsants (%)**	32		32	
**ECT (%)**	30		9	
**Comorbid ADHD (%)**	12		26	
**Comorbid anxiety disorder (%)**	28		27	
**Alcohol abuse (%)**	24		23	
**Substance abuse (%)**	9		25	

The controls (n = 86) were matched for age and sex (X% female). No differences were found regarding education level between the bipolar disorder groups and the control group.

^a^ data from 47–64 patients

^b^ data from 36–44 patients

The age- and sex-matched healthy, population-based control subjects (*n* = 86) were randomly selected by Statistics Sweden (SCB). The screening for past or present psychiatric disorders was performed using M.I.N.I. [18]. The exclusion criteria for healthy controls have been described in detail previously [25].

Some parts of the neurocognitive data from patients and controls enrolled in this study have been used in a previous study that used univariate analysis to compare cases and controls [6].

### Neuropsychological test procedure

Participants were assessed with 21 tests, most of which are described in detail by Lezak et al., [26] tapping key aspects of cognition, including executive function and attention, processing speed, memory and verbal skills. The battery usually required two sessions with patients, whereas the controls were assessed during a single session. The following tests were used.

*The Claeson-Dahl Verbal Learning and Retention Test* is a word list learning task that presents 10 words for a maximum of ten learning trials. Measures of importance are the learning score, the retention score, and the recognition score.

Five stand-alone tests from the *Delis-Kaplan Executive Function System (D-KEFS)*: the *Color-Word Interference Test* (condition 1: Color Naming, condition 2: Word Reading, condition 3: Inhibition, condition 4: Inhibition/Switching), the *Design Fluency Test* (condition 1: Filled Dots, condition 2: Empty Dots Only, condition 3: Switching, the *Tower Test* (total achievement Score and total rule violations), the *Trail Making Test* (condition 1: Visual Scanning, condition 2: Number Sequencing, condition 3: Letter Sequencing, condition 4: Number-Letter Switching and condition 5: Motor Speed), the *Verbal Fluency Test* (condition 1: Letter fluency, condition 2: Category Fluency and condition 3: Category Switching).

All tests comprising the *Wechsler Adult Intelligence Scale* (WAIS-III), except the Object Assembly Test.

The *Continuous Performance Test II* (CPT-II) with number of commission and omission errors and reaction time.

The *Rey Complex Figure Test* (RCFT) with measures of immediate and delayed recall plus recognition.

### Statistical procedures

For each cognitive measure, skewness and kurtosis were determined and then when appropriate data were transformed according to the ladder of powers. The direction of the results was adjusted such that high scores represented good performance. Following unit variance scaling and mean-centering, data were modeled by means of OPLS-DA, implemented by SIMCA-P 13.0 software (Umetrics AB, Umeå, Sweden). The OPLS-DA procedure identifies correlation patterns that discriminate between pre-defined groups and assesses the relative importance of each test variable for the discrimination [13]. Furthermore, conventional PLS calculations were performed, which apply to the two-block regression problem. Psychosocial and clinical variables (e.g., MADRS scores, number of depressions) were used to construct models of cognitive performance (47 neuropsychological measures) in patients.

## Results

Table 1 shows demographic and clinical characteristics for the bipolar disorder group and the control group. Premorbid IQ as assessed by years of education was not significantly different between the bipolar disorder group and the control group (F (2,193) = 1.02, p > 0.05, Partial Eta Squared = 0.01). As expected, patients with bipolar I disorder showed a higher occurrence of prior psychosis and treatment with antipsychotics than bipolar II patients.

**Figure 1:**
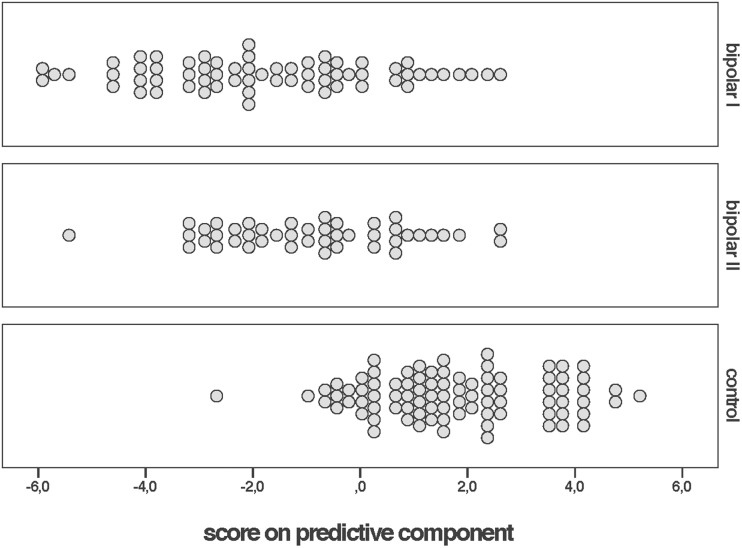
OPLS-DA score plot showing a partial separation between patients with bipolar disorder I (top panel), bipolar disorder II (middle panel) and healthy controls (lower panel). Each participant’s score is represented by a circle. The scores were t[1] values on the component predictive of diagnostic group. The vast majority (97%) of the participants were within a ±2 standard deviation limit according to Hotelling’s T^2^. Positive values represent better overall performance.

The OPLS-DA procedure yielded a model that was significant by cross-validation. The predictive component accounted for 13% of the neuropsychological variation, with a prediction ability of 0.16, according to cross validation. This indicates that the case and control groups were partially separated on the basis of their cognitive performance, the overall pattern being that the two bipolar groups performing similar and somewhat poorer than controls (Fig. 1).

The order of presentation of the individual neuropsychological measures in Table 2 reflects how well they contributed to the separation between the groups (i.e., the size of the OPLS-DA loadings). The tests with the strongest class discriminating ability were Trail Making Test 2 (number sequencing), Trail Making Test 3 (letter sequencing), Trail Making Test 4 (number-letter switching), Symbol Search, Verbal Fluency 2 (category fluency), Verbal Fluency 3 (category switching), Color-Word 3 (inhibition) and Color-Word 4 (inhibition/switching). Table 2 also presents the mean and 95% confidence interval (CI) for each measure. There was little CI overlap between bipolar patients and healthy controls on the top-loading measures, suggesting reliable case-control differences. By contrast, the CIs of the two patient groups (bipolar I and II) intersected in the vast majority of cases.

**Table 2:** Performance of euthymic patients with bipolar disorder I (BD I), bipolar disorder II (BD II), and healthy controls (C) on a neuropsychological test battery.

**Test** **and loading on the predictive component**	**BD I**	**95% Confidence Interval**	**Patients ≤ 1.25 s.d. (%)**	**BD II**	**95% Confidence Interval**	**Patients ≤ 1.25 s.d. (%)**	**Controls**	**95% Confidence Interval**	**F (2,(df))**	***p***	**Effect Size**	**Post Hoc result at statistically significant difference**
Trail Making Test 4^a^ **0.27** ^b^	9.0	8.4–9.6	48	9.4	8.7–10.2	43	11.6	11.1–12.6	24.2(141)	0.000	.20	BD I,BD II < C
Verbal Fluency 3^a^ **0.25** ^b^	11.0	10.2–11.9	34	11.5	10.5–12.5	27	13.6	12.9–14.4	12.12(186)	0.000	0.13	BD I,BD II < C
WAIS-III: Symbol Search **0.24** ^b^	9.7	9.1–10.4	28	10.2	9.4–11.0	19	11.9	11.4–12.5	13.9(189)	0.000	0.13	BD I,BD II < C
Trail Making Test 2^a^ **0.24** ^b^	8.6	7.9–9.3	34	10.1	9.2–11.0	19	11.5	10.8–12.1	16.9(186)	0.000	0.15	BD I,BD II < C^c^
Verbal Fluency 2^a^ **0.23** ^b^	12.4	11.4–13.3	38	11.7	11.2–13.4	39	15.98	15.2–16.8	21.0(186)	0.000	0.18	BD I,BD II < C
Verbal Fluency 3-v^a^ **0.23** ^b^	11.4	10.6–12.2	18	11.7	10.8–12.7	11	13.4	12.7–14.1	8.6(188)	0.000	0.08	BD I,BD II < C
Trail Making Test 3^a^ **0.22** ^b^	9.2	8.5–9.9	22	9.8	8.9–10.7	14	11.1	10.5–11.7	8.5(186)	0.000	0.08	BD I,BD II < C
Color Word 3^a^ **0.22** ^b^	9.6	8.8–10.3	29	9.9	9.0–10.8	29	11.7	11.0–12.3	10.5(181)	0.000	0.10	BD I,BD II < C^c^
Color Word 4^a^ **0.21** ^b^	9.4	8.7–10.2	27	10.1	9.2–11.0	15	11.3	10.7–11.9	7.0(180)	0.000	0.08	BD I,BD II < C^c^
Design Fluency 3^a^ **0.21** ^b^	11.0	10.3–11.6	32	11.6	10.8–12.4	17	13.1	12.5–13.6	13.10(184)	0.000	0.11	BD I,BD II < C
WAIS-III: Digit Symbol—Coding **0.20** ^b^	9.0	8.3–9.7	19	9.7	8.8–10.5	11	11	10.35–11.6	8.93(190)	0.000	0.09	BD I,BD II < C
RCFT: Time to copy^a^ **0.19** ^b^	234	205–263	23	200	165–234	24	160	135–185	7.40(180)	0.001	0.08	BD I,BD II < C^c^
WAIS-III: Block Design **0.18** ^b^	11.0	10.3–11.8	40	12.2	11.4–13.1	44	13.1	12.5–13.8	8.80(190)	0.000	0.08	BD I,BD II < C^c^
Tower: Total^a^ **0.18** ^b^	10.6	9.7–11.4		11.1	10.12–12.1		11.9	11.1–12.7	2.80(150)	0.07	0.03	-
WAIS-III: Letter-Number Sequencing **0.17** ^b^	9.7	9.1–10.5	22	9.3	8.5–10.2	28	11.2	10.6–11.8	8.02(188)	0.000	0.08	BD I,BD II < C
WAIS-III: Symbol Coding-Copy **0.17** ^b^	106	100–111	35	115	108–123	19	119	113–125	5.40(123)	0.006	0.08	BD I < BD II, C^c^
WAIS-III: Digit Symbol-Coding- Free Recall **0.16** ^b^	7.2	6.8–7.6	25	7.4	7–7.9	16	8.0	7.7–8.4	5.20(144)	0.006	0.07	BD I < BD II, C^c^
Color Word 1^a^ **0.15** ^b^	8.7	8.0–9.3		9.1	8.3–9.9		9.8	9.2–10.4	2.94(181)	0.056	0.03	-
RCFT: Recognition **0.14** ^b^	42.5	39.4–45.6	22	44.5	40.9–48.1	21	50.2	47.5–52.8	7.70(178)	0.001	0.08	BD I,BD II < C
WAIS-III: Arithmetic **0.14** ^b^	10.5	9.9–11.1	37	10.6	9.8–11.3	31	12.0	11.5–12.6	8.30(186)	0.000	0.08	BD I,BD II < C
CPT: Omission errors^a^ **0.14** ^b^	57.5	48–66		48.3	38–58		42.3	34.8–49.6	3.54(191)	0.031	0.04	-^c^
WAIS-III: Matrix Reasoning **0.13** ^b^	12.5	11.8–13.1	21	12.4	11.6–13.1	30	13.52	13–14.1	4.30(190)	0.015	0.04	-
WAIS-III: Picture Completion **0.12** ^b^	10.1	9.4–10.8	14	10.5	9.7–11.3	16	11.3	10.7–11.9	3.80(190)	0.023	0.04	BD I < BD II, C
Verbal Fluency 1^a^ **0.12** ^b^	12.2	11.2–13.1		12.4	11.2–13.5		13.7	12.9–14.6	3.56(190)	0.03	0.04	-
WAIS-III: Digit Symbol-Paired	12.1	10.9–13.2	26	13.1	11.7–14.4	27	14.4	13.3–15.5	4.20(142)	0.02	0.05	BD I < BD II, C
WAIS-III: Similarities **0.11** ^b^	10.6	9.8–11.4	13	10.5	9.5–11.4	11	12.4	11.7–13.1	7.50(189)	0.001	0.07	BD I,BD II < C^c^
RCFT: 3 min recall^a^ **0.11** ^b^	41.2	37.7–44.7	23	44.4	40.1–48.0	21	48.4	45–51	4.80(186)	0.001	0.05	BD I < BD II, C
Tower: Rule Violations^a^ **0.10** ^b^	1.29	0.6–2.0	34	1.12	0.3–2.0		0.3	-0.4–1.0	2.20(153)	0.111	0.03	BD I < BD II, C
CPT: Comission Errors^a^ **0.10** ^b^	53.6	51.0–56.0		56.3	53.0–59.0		51.4	49–54	3.00(170)	0.05	0.03	-
WAIS-III: Digit Span Backward **0.10** ^b^	4.8	4.4–5.1		5.0	4.6–5.4		5.3	5.0–5.6	2.90(189)	0.05	0.03	-
Claeson Dahl^a^ Recognition **0.09** ^b^	9.6	9.4–9.8		9.7	9.5–9.7		9.8	9.6–9.9	1.00(179)	0.36	0.01	-
WAIS-III: Digit Span^a^ **0.09** ^b^	9.5	8.8–10.2		9.7	8.9–10.6		10.6	10.0–11.2	3.10(190)	0.047	0.03	-
RCFT: 30 min recall^a^ **0.08** ^b^	43	39.4–46.6		43	38.8–47.3		46.8	43.9–49.9	1.73(183)	0.18	0.02	-
Design Fluency 1^a^ **0.08** ^b^	11.2	10.5–11.9		11.2	10.3–12.1		11.74	11.1–12.3	0.80(184)	0.452	0.01	-
WAIS-III Picture Arrangement **0.08** ^b^	9.6	8.9–10.4		10.7	9.9–11.7		10.8	10.2–11.4	3.20(189)	0.044	0.03	-
CPT: Reaction time^a^ **0.08** ^b^	390	373–407		357	337–377		361	346–376	4.30(173)	0.015	0.048	-^c^
Claeson Dahl Learning^a^ **0.07** ^b^	46.6	43.8–49.3		47.5	44.3–50.1		49.3	46.9–51.6	1.17(186)	0.312	0.012	-
Color Word 2^a^ **0.07** ^b^	9.7	9.0–10.3		10.4	9.7–11.2		10.1	9.6–10.6	1.28(181)	0.289	0.014	-
Claeson Dahl Retention^a^ **0.07** ^b^	47.3	44.0–50.3		44.3	40.6–47.6		48.0	45.5–50.6	1.60(187)	0.197	0.017	-
Design Fluency 2^a^ **0.06** ^b^	11.2	10.5–11.9		11.1	10.9–12.1		11.5	10.9–12.1	0.43(184)	0.650	0.005	-
WAIS-III: Digit Span Forward **0.06** ^b^	6.3	6.0–6.5		6.1	5.8–6.5		6.4	6.2–6.7	1.00(189)	0.36	0.01	-
Trail Making Test 1^a^ **0.05** ^b^	10.5	9.9–11.1		11.1	10.4–11.8		10.9	10.2–11.4	0.75(186)	0.47	0.01	-
Trail Making Test 5^a^ **0.02** ^b^	11.4	10.9–11.9		11.7	10.0–12.3		11.8	11.4–12.3	0.78(186)	0.46	0.01	-
WAIS-III: Information-**0.02** ^b^	13.2	12.6–13.8		13.6	12.9–14.2		13.4	12.9–13.8	0.30(188)	0.72	0.00	-
WAIS-III: Comprehension-**0.03** ^b^	11.4	10.7–12.2		11.4	10.5–12.3		11.8	10.93–12.7	0.20(128)	0.80	0.00	-
WAIS-III: Vocabulary-**0.05** ^b^	11.5	10.9–12.1		11.3	10.6–12.0		11.3	10.8–11.8	0.20(190)	0.82	0.00	-
RCFT: Copy^a-^ **0.08** ^b^	33.6	32.8–34.4		32.7	32.8–33.6		32.7	32.0–33.3	1.91(186)	0.15	0.00	-

The neuropsychological measures are arranged according to the size of the OPLS-DA loadings. Results are expressed as means, 95% confidence intervals (CIs) and effect sizes (*η* ^2^). Percentage was calculated of patients scoring ≤ the 1.25 s.d. of the control group.

*Note.*

^a^ Pålsson et al, 2012,

^b^ loading on predictive component,

^c^ Games Howell otherwise Scheffé.

Table 2 also shows that the proportion of patients whose performance fell 1.25 standard deviations below controls was sizeable on the top-loading tests. For instance, 48% of patients with bipolar I disorder and 43% of patients with bipolar II disorder fulfilled this criterion for impairment on Trail Making Test 4.

A series of PLS models were created using patient data in order to assess the extent to which clinical and psychosocial variables could be understood in terms of cognitive performance. Regressing MADRS and YMRS scores separately against cognition yielded non-significant models, suggesting that the neuropsychological performance was not related to mood within the present scale interval (i.e., MADRS/YMRS scores below 14). Likewise, non-significant models were obtained when cognitive performance was regressed against GAF and CGI scores, the number of affective episodes, ADHD co-morbidity, a history of psychosis, or number of sick-leave days. Non-significant models were also obtained when regressing ongoing drug treatment (antidepressants, antipsychotic medication expressed as CPZ equivalents, lithium, and anticonvulsants) to cognitive performance in patients.

## Discussion

Neuropsychological test batteries generate large datasets with many variables that are challenging to summarize and evaluate using traditional statistical tools that primarily are designed to analyze ‘long-and-lean’ data tables [13]. Some of the between subject variation in performance on a broad cognitive battery is shared by all tests, therefore the performance on a single test is related to latent mental ability required to perform all tests [27]. Statistical tools developed to deal with large datasets with inter-correlated measures in other research areas may therefore be useful for the study of cognition in mental illness. Here, we employed the OPLS-DA to sift the results from 21 neuropsychological tests, yielding 47 performance measures, in patients with bipolar disorder and healthy controls, the aim being to identify specific tasks that detect cognitive weaknesses in the patient group.

As applied here, OPLS-DA partitioned the variation in neuropsychological performance into two components, one being associated with group membership and the other irrelevant. When examining the explained variance in more detail it was apparent that only measures from nine tests defined the class separation: Trail Making Test, Verbal/Design Fluency, Color-Word, Tower Test, Symbol Search, Block Design, Letter-Number Sequencing, and Symbol Coding. The remaining 12 tests, including measures of semantic knowledge, verbal learning and some tests of working memory/attention, did not tell the groups apart.

The combination of nine identified tests may hence be considered for identifying cognitive impairment in bipolar patients in research, and possibly in a clinical setting as well. The set of tests identified here are with some exceptions the same as, or similar to, the ones recommended for inclusion in a battery for cognition in bipolar disorder by the International Society for Bipolar Disorder (ISBD) on the basis of meta-analyses [12]. For instance, the usefulness of the Trail Making Test A, Letter-Number Sequencing, Category Fluency and Digit Symbol Coding was confirmed in our study. Moreover, our analysis revealed the importance of TMT 4 (similar to TMT B) and Color-Word Interference Test, which are in line with the result from a recent evaluation of the clinical efficacy of the tests recommended by ISBD [28].

However, some of the tests proposed by Yatham et al. [12] received relatively low loadings in the present study and did not differentiate between patients and controls. These included tests for attention/vigilance (Continuous Performance Test), verbal learning and memory (Claeson-Dahl Verbal Learning and Retention Test), and visual learning (most measures of the Rey Complex Figure Test). Our analysis reveals that some of the tests suggested by Yatham et al. [12] might be redundant, being unable to differentiate between patient and controls. It could also be that patients with problems in these domains may constitute a cognitive subgroup that was insufficiently represented to gain statistical significance in the present sample.

It should be noted that the tests used in present study are diagnostically non-specific. For example, deficits on the Trail Making Test have been observed in conditions as disparate as posttraumatic stress disorder [29] and myotonic dystrophy type 1 [30]. Nevertheless, the tests do go some way in directing researchers’ attention to particular cognitive domains such as, in the present case, searching, fluency, switching and inhibition, i.e., processes with a distinct ‘prefrontal’ flavour, which could be specified further using more fine-grained cognitive instruments.

As to the question whether the cognitive impairment in bipolar disorder is clinically significant, inspection of the group means and their CIs for the nine discriminatory tests confirmed their usefulness for identifying impairments in the patient groups. For a substantial minority of the patients the impairments approached clinical significance (as defined by a performance 1.25 SD below controls) on certain tests (e.g., for >40% on Trail Making Test 4 and Block Design tests). Such findings may be rooted in the fact that bipolar disorder is associated with palpable changes in brain structure, such as ventricular enlargement and shrinkage of medial temporal lobe areas [31]. A reduction in temporal lobe gray matter has been associated with decline in intellectual function and with numbers of mood episodes in patients with bipolar disorder [32]. However, we emphasize that the OPLS-DA-aided group separation was partial and incomplete, with non-trivial overlap in the overall cognitive performance between patients and controls (see Fig. 1). Hence, the observed group differences could not be used for predicting diagnoses on an individual level.

We investigated whether the subtypes of bipolar disorder differ in neuropsychological terms but found that patients with bipolar I disorder were indistinguishable from patients with bipolar II disorder on 16 of the 18 cognitive measures (89%) with discriminant loadings ≥ 0.15. This correspond to the results from our own group where we used measures of verbal and visual memory test and executive function to test this hypothesis using univariate statistics in the same cohort, and also the findings by a previous study by Dittman et al. [5], but contrasts with the results showing that bipolar I patients have more severe cognitive dysfunction compared with patients with bipolar II [7–9]. By and large, the cognitive impairments seen in patients with bipolar I and bipolar II disorder are hard to differentiate and appear to have much in common [5, 6].

Bipolar disorder can take on psychotic manifestations requiring antipsychotic medication, and is commonly accompanied with excessive alcohol/drug use [33] and psychosocial and occupational problems [34]. Difficulties of this nature were observed also in the present patient group (see Table 1) and might conceivably have contributed to the observed cognitive deficits [35, 36]. Our attempts to model these relationships showed that medication alone could not explain the variance in cognitive performance, which was somewhat surprising given that our own group showed that treatment with antipsychotics was associated with worse performance on the time to draw parameter of the Rey complex figure test, number sequencing, letter sequencing and number-letter switching conditions of the Trail making test and all trials of the Verbal fluency test [6]. An explanation might be that the multivariate modelling creates a new summarizing variable, which captures a latent structure in cognitive performance and is somewhat different from the original variables. However, results in the current study are in line with re-analysis of earlier studies suggesting that most neuropsychological tests do not exhibit any significant association with ongoing pharmacological treatment [10].

As to the other clinical and psychosocial background factors (viz. co-morbidity with ADHD, substance/alcohol abuse, GAF scores, number of affective episodes or sick-leave days), the associations were not stable enough to gain statistical significance. The modest associations with everyday occupational and social factors may suggest that the tests used here are of questionable ecological validity [37]. Moreover, a meta-analysis, investigating the association between cognitive ability and everyday functioning in bipolar disorder showed results similar to that seen in schizophrenia, with small differences across cognitive domains [38]. The strength of association differed to a greater extent with respect to the functional measurement approach. Nevertheless, the lack of associations in current study are consistent with a recent re-analysis of studies in the field showing that the majority of cognitive measures were not associated with measures of illness severity [10].

By including patients scoring between 0 and 13 on the MADRS/YMRS scales, the criterion for defining euthymia in the present study was more liberal than in earlier studies [1]. This raises the possibility that residual mood symptoms significantly affected performance in the cognitive tasks [39]. However, the shared variance between the mood scale values and overall cognitive performance was not sufficiently stable to generate a valid statistical model. In line with previous studies, [2, 40, 41] we conclude that mood have limited value in explaining the cognitive impairments in euthymic bipolar patients.

The strengths of the current study are that patients are representative of bipolar patients that receive psychiatric care. At the time of enrollment, virtually all patients with bipolar disorder in the Northern Stockholm catchment area were referred to the Affective unit for work up and treatment. Moreover, the diagnoses have been made with a best-estimate procedure by experienced clinicians specialized in bipolar disorder. The neuropsychological test battery was administered under standardized conditions. Controls were randomly selected from the population in the same catchment area and matched for sex and age rather than being university students or health care workers. A limitation to consider, however, is that information on drug use was not collected on the day of testing, but from either the baseline examination or the day of blood sampling. On the other hand, enrolled patients were in a stable euthymic mood and the overwhelming majority of patients would not have added or discontinued medication between these occasions.
